# TMBur: a distributable tumor mutation burden approach for whole genome sequencing

**DOI:** 10.1186/s12920-022-01348-z

**Published:** 2022-09-07

**Authors:** Emma Titmuss, Richard D. Corbett, Scott Davidson, Sanna Abbasi, Laura M. Williamson, Erin D. Pleasance, Adam Shlien, Daniel J. Renouf, Steven J. M. Jones, Janessa Laskin, Marco A. Marra

**Affiliations:** 1 Canada’s Michael Smith Genome Sciences Centre, BC Cancer, Vancouver, BC Canada; 2 Program of Genetics and Genome Biology, The Hospital for Sick Children, The Peter Gilgan Centre for Research and Learning, Toronto, ON Canada; 3 Department of Medical Oncology, BC Cancer, Vancouver, BC Canada; 4 Department of Medical Genetics, University of British Columbia, Vancouver, BC Canada

**Keywords:** Tumor mutation burden, Whole genome and transcriptome analysis (WGTA), Immune checkpoint inhibitors

## Abstract

**Background:**

Tumor mutation burden (TMB) is a key characteristic used in a tumor-type agnostic context to inform the use of immune checkpoint inhibitors (ICI). Accurate and consistent measurement of TMB is crucial as it can significantly impact patient selection for therapy and clinical trials, with a threshold of 10 mutations/Mb commonly used as an inclusion criterion. Studies have shown that the most significant contributor to variability in mutation counts in whole genome sequence (WGS) data is differences in analysis methods, even more than differences in extraction or library construction methods. Therefore, tools for improving consistency in whole genome TMB estimation are of clinical importance.

**Methods:**

We developed a distributable TMB analysis suite, TMBur, to address the need for genomic TMB estimate consistency in projects that span jurisdictions. TMBur is implemented in Nextflow and performs all analysis steps to generate TMB estimates directly from fastq files, incorporating somatic variant calling with Manta, Strelka2, and Mutect2, and microsatellite instability profiling with MSISensor. These tools are provided in a Singularity container downloaded by the workflow at runtime, allowing the entire workflow to be run identically on most computing platforms. To test the reproducibility of TMBur TMB estimates, we performed replicate runs on WGS data derived from the COLO829 and COLO829BL cell lines at multiple research centres. The clinical value of derived TMB estimates was then evaluated using a cohort of 90 patients with advanced, metastatic cancer that received ICIs following WGS analysis. Patients were split into groups based on a threshold of 10/Mb, and time to progression from initiation of ICIs was examined using Kaplan–Meier and cox-proportional hazards analyses.

**Results:**

TMBur produced identical TMB estimates across replicates and at multiple analysis centres. The clinical utility of TMBur-derived TMB estimates were validated, with a genomic TMB ≥ 10/Mb demonstrating improved time to progression, even after correcting for differences in tumor type (HR = 0.39, *p* = 0.012).

**Conclusions:**

TMBur, a shareable workflow, generates consistent whole genome derived TMB estimates predictive of response to ICIs across multiple analysis centres. Reproducible TMB estimates from this approach can improve collaboration and ensure equitable treatment and clinical trial access spanning jurisdictions.

**Supplementary Information:**

The online version contains supplementary material available at 10.1186/s12920-022-01348-z.

## Background

Tumor mutation burden (TMB) is a somatic characteristic that can reveal underlying mechanisms of tumor progression, as well as inform on prognosis [1] and potential response to immune checkpoint inhibitors (ICIs) [2, 3]. Although response rates to ICIs in unselected populations are low (5–13% overall response rates to PD-1 inhibitors) [4, 5], rates increase when patients are selected based on tumor characteristics, such as high TMB [6] (≥ 10/Mb, 41% objective response to PD-1/PD-L1 inhibitors).

Despite the continuing proliferation of cancer sequencing activities, the only FDA-approved TMB companion diagnostic is the FoundationOne® CDx panel [7, 8]. Previous studies have reported the variability of TMB estimates derived from panel approaches, indicating they may not accurately reflect whole-genome TMB [9, 10]. Whole exome sequencing is routinely performed at many research institutes. However, the resulting biased coverage profile affects mutation calling [11] and thus TMB and mutation signature analyses. Whole genome sequencing addresses this and related issues.

Earlier studies have shown that for whole genome sequence data, differences introduced through extraction or library construction methods are far outweighed by differences introduced in analysis methods [12]. Enrollment of patients in multicentre trials is an example in which the application of a consistent analysis pipeline would benefit study quality. While small differences in TMB values may not appear to be problematic, they can significantly impact patient selection for ICI trials where a threshold of 10 mutations per Mb has been clinically approved [13]. For example, taking orginally published TMB values [2], the application of the TMBur pipeline would result in a change in eligibility (based on 10 mutations per Mb) for two patients (2.5%, total n = 82).

To address the problem of variability between analysis pipelines deployed at distinct institutions (Additional file 1: Table S1), we present a TMB estimation pipeline, TMBur, purposefully designed to be shared among centres. TMBur performs end-to-end analysis of tumor and matched normal whole genome sequence data to provide TMB counts for whole genomes and subsets to coding space, protein modifying alterations, and pseudo-panels.

## Methods

### Sequencing

Tumor specimens were collected from biopsies (needle core, endobronchial ultrasound) or tissue resections (Additional file 2: Table S2). Solid tumor specimens were snap frozen and liquid biopsies were spun down. Pathology was reviewed and nucleic acids were extracted as described in Pleasance et al. [1]. Sequencing was performed on either HiSeq 2500 or HiSeq X instruments to target 80X coverage for the tumor samples and 40X coverage for the matched normal samples. The reference cell lines COLO829 and COLO829BL were obtained from American Type Culture Collection (ATCC), Manassas, VA, and sequenced to 80X and 40X coverage, respectively.

### TMBur pipeline

The TMBur pipeline, implemented in Nextflow [14], performs all analysis steps to generate TMB estimates from fastq files, including adapter trimming with fastp [15], alignment with BWA mem 0.7.17 [16], and alignment sorting and duplicate marking with Samtools 1.9 [17]. Somatic variants are identified using Manta 1.6.0 [18], Strelka 2.6.2 [19], and Mutect2 from GATK 4.0.10.0 [20]. Variants from these tools are intersected using RTGTools [21] to generate the calls used for further analysis. Microsatellite instability (MSI) is estimated using MSIsensor2 0.1 [14], while all annotation of variants is done against Ens75 [22] using SNPEff 4.3t [23]. Intersections of coordinate ranges are performed using bedtools 2.29.2 [24]. These tools are provided in a singularity 3.5.2–1.1.el7 [25] container downloaded by the workflow at runtime.

Calculation of TMB for somatic variant genomic subsets is as follows:

- *Genome* counts of variants that overlap with chromosomes 1-22XY are divided by the alignable space (non-N bases) in 1-22XY (n = 2667837836 bases).
- *Coding* counts of variants that intersect with CDS bases from Ens75 are divided by the total CDS bases in 1-22XY (n = 31990128 bases).
- *Protein* counts of variants with SnpEff impact rating of “HIGH” or “MODERATE” using Ens75 are divided by the total CDS bases in 1-22XY.
- *Panel* using methods outlined in [26], the count of variants is limited to the CDS of a set of cancer genes (Additional file 3: Table S3) minus: (a) any variants intersecting with COSMIC [27] (b) nonsense SNVs and SNPEff impact ratings of “HIGH”, “MODERATE”, or “LOW” in tumor suppressor genes (Additional file 4: Table S4).

Variant calling using TMBur is stable down to a normal depth of 30X, and a tumor depth of 50X with bioinformatics estimated tumor content values above 30%, based on tests performed using Mutect2 and Strelka2, components of TMBur (Additional file 8: Fig. S1).

### Reproducibility at multiple sites

Analysis infrastructure at Canada’s Michael Smith Genome Sciences Centre at BC Cancer in Vancouver, Canada and The Hospital for Sick Children in Toronto, Canada, used CentOS 7 on Intel-based CPUs. Vancouver’s cluster scheduling was done with slurm, and Toronto’s was performed with Moab/Torque (Additional file 5: Table S5).

### Clinical data collection and survival outcomes

The ICI-treated patient cohort’s complete treatment histories, response, and survival data were collected retrospectively using the BC Cancer Pharmacy database and chart review, and as described in Pender et al. [2]. Patients received either a single-agent ICI, combination ICIs, or a combination of ICI and chemotherapy. Follow-up was censored on March 1, 2019. Time to progression (TTP) was defined as the time from ICI initiation to the date of discontinuation due to progression.

Kaplan–Meier survival analysis was performed for TTP using the R packages survival (v3.1-8) and survminer (v0.4.7). For all survival analyses, patients were split into high and low groups based on a threshold of 10/Mb. Log-rank tests were used to calculate *p* values. Cox proportional hazards models were performed on 78 samples using the R packages survival (v2.42.3) and forest model (v0.5.0) individually for each TMB estimate combined with tumor type. Tumor types were only included if at least three patients with that tumor type were available. Counts from indel-only groups and MSI scores were excluded from these analyses, as the 10/Mb threshold was not appropriate.

## Results and discussion

**Fig. 1:**
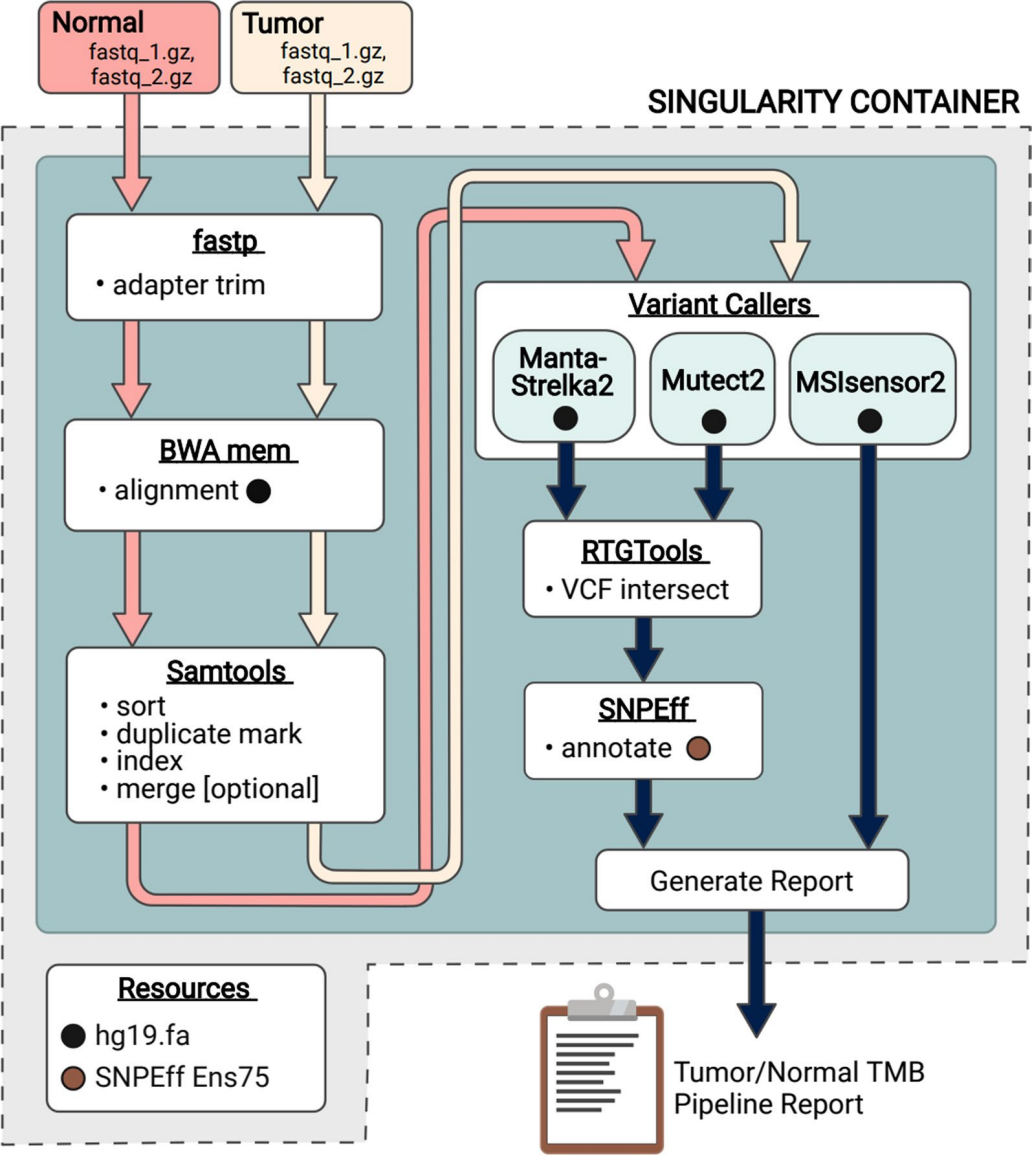
Schematic diagram of the Nextflow workflow for the singularity container used in the TMBur pipeline. TMBur is a portable software package that contains multiple individual components, including variant caller tools (Manta-Strelka2, Mutect2, and MSIsensor2) and resources (the human genome reference sequence [hg19] and reference annotations [SNPEff Ens75]), all used to provide consistent tumor mutation burden (TMB) counts. The workflow allows for the analysis of raw, whole-genome data from pairs of samples (tumor and normal) and can be conducted with multiple pairs simultaneously

The TMBur workflow generates TMB estimates directly from fastq files, handling both alignment and variant calling (Fig. 1). TMB estimates can be generated for matched 80X tumor and 40X normal genomes in 24 h on a computer cluster with 1.5 Tb of storage, 96 Intel(R) Xeon(R) CPU E5-2650 v4 @ 2.20 GHz CPUs, and 600 GB of RAM. If desired, intermediate BAM and VCF files can be saved for review or further analysis.

Reproducibility tests were performed with TMBur 2.2.5 by repeatedly analyzing whole genome data from the somatic reference cell line COLO829 (COLO829BL normal). All TMB (per Mb) estimates reported by TMBur were identical across 10 analysis replicates. Small single digit variances in the genome-wide SNV (range 38014–38020) and INDEL counts (1301–1302) were observed (Additional file 6: Table S6). Upon investigation, this variance was attributed to the pseudo-random read placement employed by BWA [28]; multiple runs using the same starting fastqs yielded BAM files that had 0.2% of alignments with differing coordinates. All employed variant callers, including Strelka2, Mutect2, MSISensor and Manta, were shown to call identical sets of variants when supplied with identical BAM files. MSI status was called stable (MSS) for each test [29].

Reproducibility was also tested using COLO829 and COLO829BL data at two centers (in Vancouver, BC and Toronto, ON) that ran TMBur using their local storage and compute infrastructures. Results of these tests showed identical TMB estimates, and the variances in genome-wide SNV (range 38014–38022) and INDEL counts (1301–1302) were similar to those reported above for the single center replicates (Additional file 6: Table S6).

**Fig. 2:**
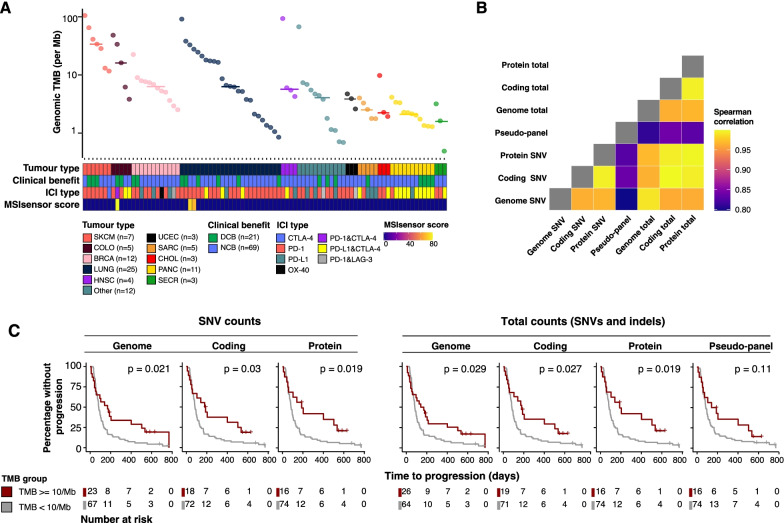
Predictive value of TMB estimates on ICI response. **A** Tumor types represented in the cohort, and genomic SNV TMB estimates per sample. **B** Spearman correlation between TMB estimates derived from different genomic subsets. **C** Time to progression in patients with a high (≥ 10) or low (< 10) TMB. *p* Values were determined using a log-rank test. In all panels, SNV counts describe TMB derived using SNVs only, and total counts refer to SNV and indels. TMB estimates are as described in Methods. Patients at risk for progression at each time point are shown in the tables below

To demonstrate the application of TMBur to a well-studied cancer patient cohort, we applied it to whole genome data from 90 patients with advanced and metastatic cancer [1, 2] who subsequently received ICIs. This cohort included 19 tumor types, with the most common being lung (n = 24, 27%), breast (n = 11, 12%), and pancreatic cancers (n = 11, 12%, Fig. 2A, Additional file 2: Table S2). Patients received an average of three cancer therapies (median, range: 1–10) prior to biopsy.

Tumor types with the highest genomic SNV TMB reported by TMBur were consistent with previous reports [26, 30], with some cutaneous melanomas, colorectal and lung cancers exhibiting the highest counts (Fig. 2A). All TMB counts (SNV only and total counts including indels, for different genomic subsets) were strongly correlated (mean R = 0.911, Fig. 2B, Additional file 8: Figure S2). Interestingly, TMB estimates derived using a pseudo-panel approach (Methods) had weaker correlations with the other TMB estimates, particularly counts derived from the whole genome (SNVs alone, R = 0.797, *p* < 2.2 × 10^−16^; total including indels R = 0.810, *p* ≤ 2.2 × 10^−16^).

The FDA-approved TMB threshold for ICI treatment is 10/Mb, a value associated with hypermutation and increased response to ICIs across many studies [2, 6, 31]. Using the TMBur-derived values from different genome subsets in our data, we sought to evaluate the predictive value of this threshold. The 10/Mb threshold was able to stratify patients based on time to progression using all reported TMBur estimates to a similar degree (Fig. 2C). However, the pseudo-panel counts had a lower statistical significance (*p* = 0.11). When the 10/Mb threshold was evaluated for each TMB count in a multivariate Cox proportional hazards model with tumor type (Additional file 7: Table S7), high TMB from SNV-based estimates tended to be more effective at stratifying patients than those from total counts, including indels (protein SNV, HR = 0.31, *p* = 0.012; genome SNV, HR = 0.39, *p* = 0.012; coding SNV, HR = 0.40, *p* = 0.026). Consistent with the Kaplan–Meier analysis, pseudo-panel estimates remained the least predictive (HR = 0.53, *p* = 0.086).

TMBur estimates for seventy-eight samples (87%) were consistently called high or low, based on the 10/Mb threshold across all reported genome subsets. Fifteen samples (17%) were consistently high, 63 (70%) were consistently low, and five (6%) differed in just one of the TMB estimates. Of the five samples that were different in one estimate, one was considered high in all, but the pseudo-panel (8.26/Mb in pseudo-panel vs. median 17.12/Mb in others) had a durable clinical benefit, remaining on pembrolizumab for 578 days without progression. The other four patients were considered low in all but one method (either pseudo-panel [1/4] or genome total [3/4]). Three of these patients did not exhibit a durable clinical benefit, but one did (10.64/Mb genome total vs. median 6.57/Mb in the others), remaining on pembrolizumab for 214 days without progression. These results demonstrate that the manner in which TMB is measured can impact clinical trial eligibility and could mean that some patients who may respond could be denied enrollment.

The use of a smaller sub-set of genome variants (a “pseudo-panel”) was the least effective at predicting clinical benefit. There are limitations in comparing this to clinical panel testing, however, as in practice, whole genome sequences tend to be generated at lower levels of sequence coverage than panel sequences. Additionally, clinical panels may have variable bait efficiency resulting in variable coverage not present in whole genome data. Finally, clinical panels routinely used to align patients to trials use algorithms to filter potential germline variants as no matched normal is sequenced, a step not required using TMBur with tumor-normal pairs. Whole genome sequencing has further benefits for aligning patients with ICIs as it allows detection of specific alterations in genes such as PBRM1, LRP1B, and SMARCB1 [32–34], as well as broader information such as heterozygosity of HLA class I alleles [35]. Some studies have also demonstrated that multiple biomarkers may be more effective at predicting response [2, 3], providing further support for using whole genome sequencing instead of individual tests for each marker.

## Conclusions

Our results demonstrate that TMBur, a shareable workflow to determine TMB, serves its intended purpose to share a reproducible workflow across analysis sites. TMB estimates were consistent across multiple runs, performed at two distant academic research centers, and in a retrospective analysis appeared capable of anticipating patient responses to ICIs. TMBur could lead to higher data consistency and quality, more effective collaboration and improved access to clinical trials, ensuring patients are not excluded from participation due to cross-centre variation in TMB estimates. TMBur additionally accommodates whole genome data, which is increasingly used as a “gold standard” data type in cancer genomic analyses.

## Supplementary Information

**Additional file 1.** Variation in TMB estimates by pipeline.

**Additional file 2.** Patient and sample demographics.

**Additional file 3.** Cancer genes for which the CDS variant count is limited to.

**Additional file 4.** Tumor suppressor genes for which nonsense SNVs and SNPEff impact ratings of “HIGH”, “MODERATE”, or “LOW” variants are excluded from CDS counts.

**Additional file 5.** Analysis infrastructure at Canada’s Michael Smith Genome Sciences Centre at BC Cancer in Vancouver, and The Hospital for Sick Children in Toronto, Canada.

**Additional file 6.** Reproducibility tests performed with TMBur.

**Additional file 7.** Multivariate Cox proportional hazards models for TMB ≥ 10/Mb with tumor type.

**Additional file 8: Figure S1**. Impact of tumor fraction and sequencing depth on variant calling. F1 scores, recall and precision for SNV and indel calling for varying tumor and normal coverage, and tumor fraction. Values for variant callers Mutect2 and Strelka2 are indicated by orange and green lines respectively. **Figure S2**. Correlation between TMB estimates and predictive value of indels. Scatter plot showing the Spearman correlation between TMB estimates from subsets of the genome. Points in red show those who are not in the same threshold group (≥ 10/< 10/Mb), and the number of samples differing between each comparison is indicated in red text (top right of each square). R values are indicated in the bottom right of each square.

## Data Availability

Genomic and transcriptomic sequence datasets, including metadata for patients, are available at https://ega-archive.org/studies/EGAS00001001159 as part of the study EGAS00001001159 with accession numbers as reported in Additional file 2: Table S2. Data for most (n = 82) of the ICI treated patients is also available for download from Pender et al. [2]. COLO829 data is available under the study EGAS00001001385. The TMBur workflow and accompanying instructions are available at https://github.com/bcgsc/TMBur.

## Abbreviations

DCB: Durable clinical benefit
ICI: Immune checkpoint inhibitors
Indel: Insertion or deletion
Mb: Megabase
MSI: Microsatellite instability
NCB: No clinical benefit
PD-1: Programmed death 1
PD-L1: Programmed death ligand 1
POG: Personalized OncoGenomics
SNV: Single nucleotide variant
TMB: Tumor mutation burden
TTP: Time to progression
WGTA: Whole genome and transcriptome analysis
