# Supplementary figures and images for: TMBur: a distributable tumor mutation burden approach for whole genome sequencing
Source: BMC Med Genomics. 2022 Sep 7;15:190. doi: 10.1186/s12920-022-01348-z (PMC9450342; doi:10.1186/s12920-022-01348-z)

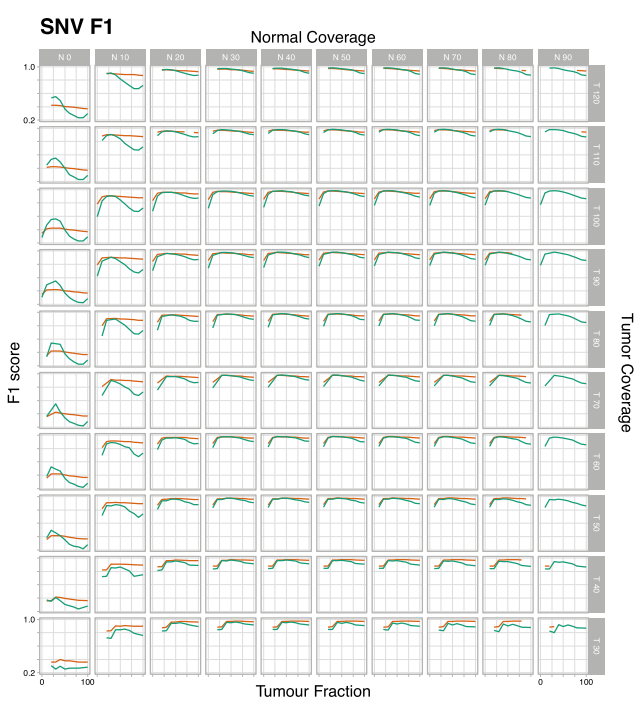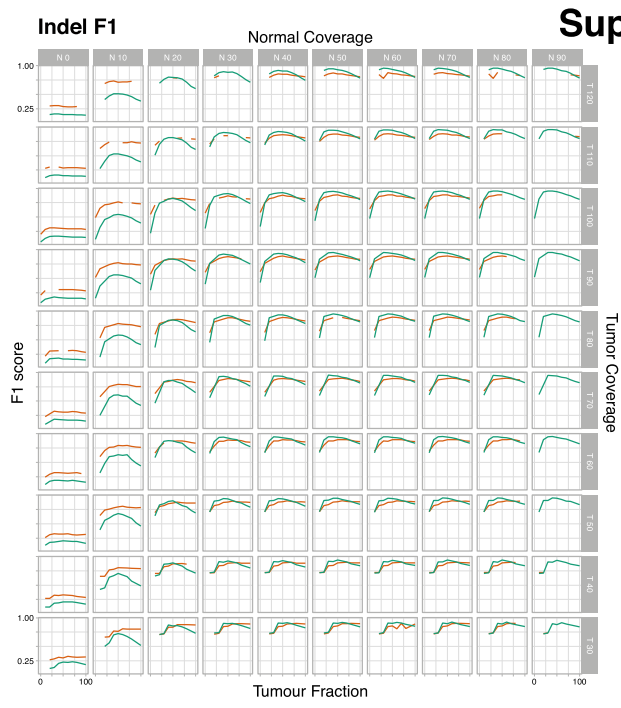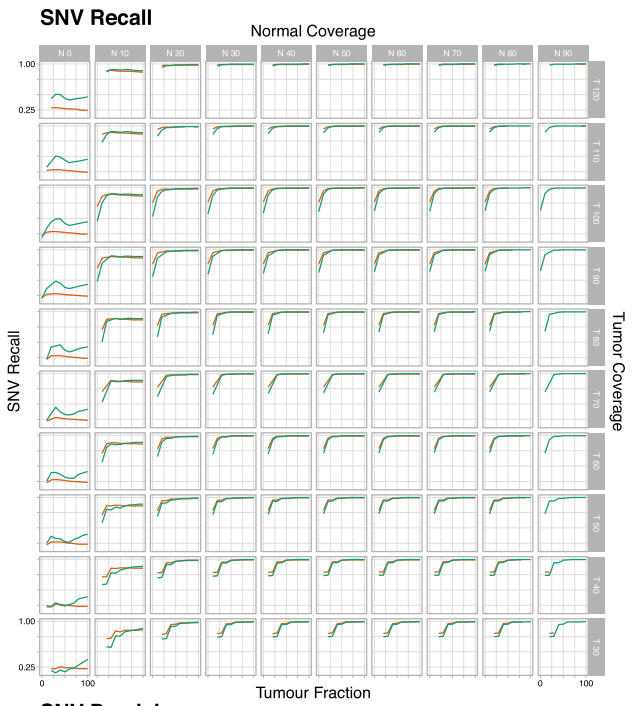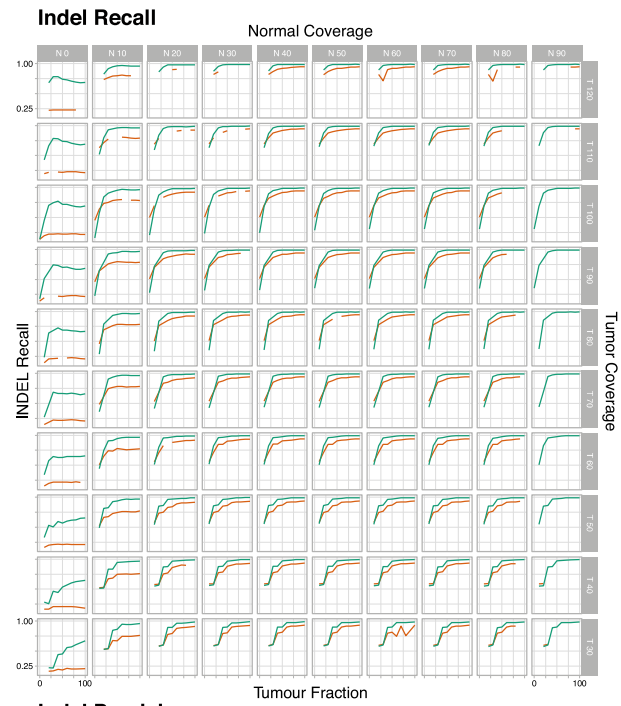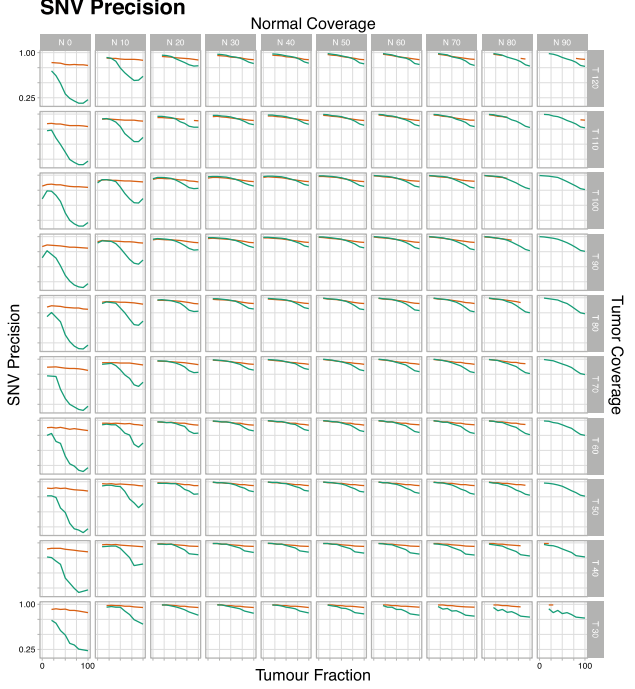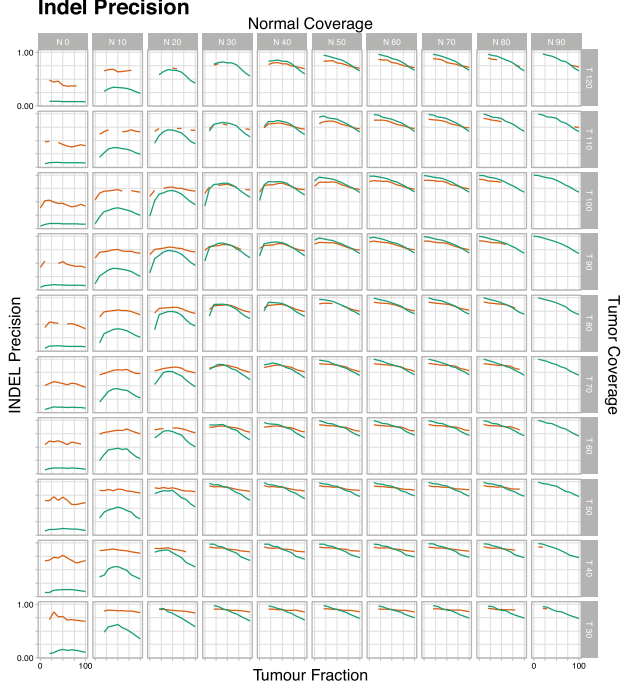

Mutation caller

Mutect2

Strelka2

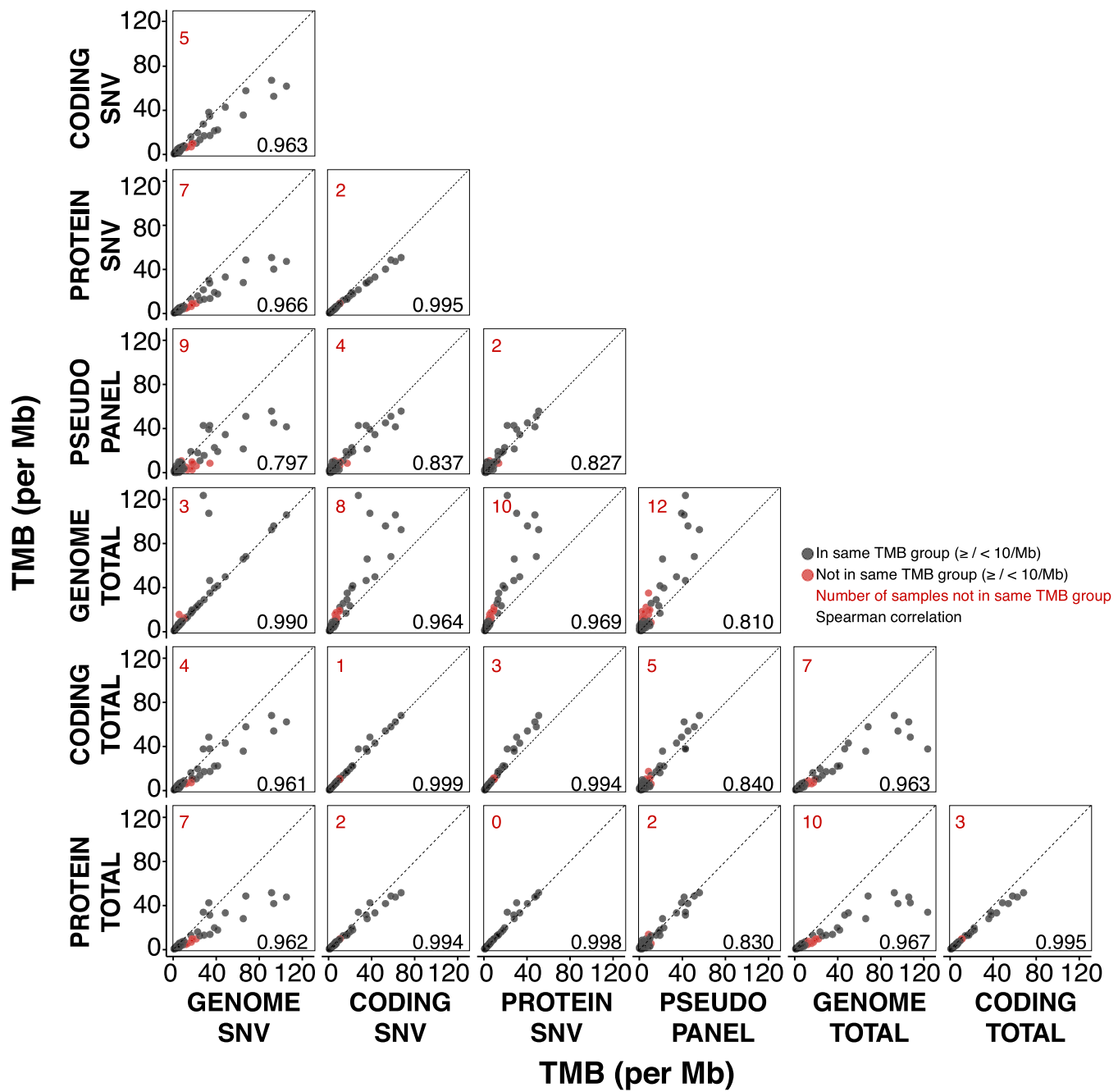

**Supplemental Figure 2**

Supplement: Supplementary file 8 — Additional file 8: Figure S1. Impact of tumor fraction and sequencing depth on variant calling. F1 scores, recall and precision for SNV and indel calling for varying tumor and normal coverage, and tumor fraction. Values for variant callers Mutect2 and Strelka2 are indicated by orange and green lines respectively. Figure S2. Correlation between TMB estimates and predictive value of indels. Scatter plot showing the Spearman correlation between TMB estimates from subsets of the genome. Points in red show those who are not in the same threshold group (≥ 10/< 10/Mb), and the number of samples differing between each comparison is indicated in red text (top right of each square). R values are indicated in the bottom right of each square. [file 12920_2022_1348_MOESM8_ESM.pdf]
